# Interprofessional Competencies for Effective Interprofessional Collaborative Practices Amongst Intensive Care Unit Teams in the North West Province, South Africa: A Cross-Sectional Survey

**DOI:** 10.1177/11786329251391944

**Published:** 2025-11-29

**Authors:** Nombulelo Esme Zenani, Suegnét Scholtz, Yolande Heymans, Christmal Christmals

**Affiliations:** 1 NuMIQ Research Focus Area, Faculty of Health Sciences, School of Nursing Science, North-West University, Mmabatho, South Africa; 2 Faculty of Health Sciences, Centre for Health Professions Education, North-West University, Potchefstroom, South Africa

**Keywords:** interprofessional competencies, interprofessional care, interprofessional collaborative practices, intensive care unit, intensive care unit teams

## Abstract

**Background:**

Intensive Care Unit (ICU) teams depend on practical interprofessional collaborative competencies to deliver comprehensive care, ensuring quality outcomes for critically ill patients. The interprofessional competency domain framework is a key strategy to support and develop attitudes, skills, values, and knowledge needed for effective interprofessional collaborative practices. This study aimed to identify the interprofessional competencies required of ICU teams in North West Province, South Africa, for effective interprofessional collaboration practices in the intensive care unit.

**Method:**

A cross-sectional study was conducted among nurses, doctors, clinical facilitators, and pharmacists working in the North West Province ICUs using a structured online self-administered questionnaire. A stratified sampling was used to recruit participants for this study. Descriptive and inferential statistics approaches were used to analyse data. Eighty-eight surveys were completed correctly and retrieved, presenting a 52% response rate of the 168 total population.

**Results:**

The findings suggest that years of experience, age, gender, and professional roles significantly impact how ICU teams perceive and execute interprofessional competencies. The study has shown that ICU teams strongly agree on the importance of all the interprofessional competencies for effective interprofessional collaborative practices in ICU settings. The interprofessional competencies promote patient-centred care, ethical conduct, team engagement, and positive team dynamics amongst the ICU teams. However, there is a need to improve at building interdependent relationships among the ICU teams. Developing interdependent relationships amongst the ICU teams will build on integrated work, communication, appreciation of each professional role, and a positive psychological environment to demonstrate interprofessional competencies. These insights underline the importance of tailored training programmes, such as interprofessional continuous professional development programmes that consider these demographic and team relations factors to enhance interprofessional collaboration practices and patient care in ICU settings.

**Conclusion:**

This study serves as a first step in determining the importance of interprofessional competencies amongst ICU teams in North West Province, South Africa, and highlights the gaps in the execution of interprofessional competencies in ICU teams. The study further provided recommendations on strategies that can be adopted to enhance the interprofessional competencies of the ICU teams. The study could set a platform for other studies that investigate interventions that could be adopted by healthcare regulatory councils, curriculum developers, and healthcare educators in developing interprofessional continuous professional development programmes for ICU teams. Interprofessional continuous professional development programmes will incorporate equity, consideration of building interdependence relations, access, and inclusion.

## Introduction

The ICU teams comprise healthcare professionals from diverse fields, including nurses, physicians, pharmacists, dieticians, physiotherapists, clinical psychologists, clinicians, and nursing trainees, each distinguished by specific roles and responsibilities.^1^ The ICU teams collectively deliver interprofessional care to critically ill patients. Interprofessional care denotes the provision of healthcare by a collaborative team of professionals possessing complementary expertise, recognising the distinct contributions of each member towards a shared objective, specifically ensuring safe and high-quality critical care in the ICU.^2,3^ Unlike other specialities, interprofessional care in the ICU demands a high level of team-based collaboration and decision-making. At times, due to the expertise available, the ICU roles may overlap or sometimes be flexible depending on the patient’s condition; therefore, this requires team understanding and clarity of roles for quality care. Team understanding and performance are critical within the low-resourced countries.

Low to middle- low-resourced countries with still maturing healthcare systems face greater challenges. These include a high burden of critical illnesses caused by factors such as urbanisation, emerging epidemics, and many people needing access to hospitals.^4-6^ Consequently, there is a higher proportion of patients presenting to emergency departments with acute illnesses, and patient outcomes tend to be worse due to limited materials, infrastructure, and trained human resources.^5^ Providing critical care in these countries is further complicated by fragmented healthcare services, poor interprofessional collaboration, and a scarcity of healthcare professionals trained in critical care, alongside concerns about relevance given competing healthcare priorities.^4-6^ Fragmented care mainly increases the risk of adverse events for critically ill patients. For example, in Ethiopia, Kifle et al^7^ reported that there are 0. 0.3 ICU beds per 100, 000 people, with only half of the ICUs providing care consistent with a level 1 ICU. Level one ICUs are designed for critically ill patients with less complex conditions, such as post-surgical patients or those with uncomplicated medical issues requiring special observation.^8^ Kifle et al^7^ also note that resources are limited: no site has piped oxygen, invasive monitoring is rarely available, isolation policies are neither documented nor implemented, and there is no established interprofessional team handover practices. The absence of these elements results in disjointed, task-oriented care and poor patient outcomes. Biccard et al^8^ identify many reasons for the limited critical care resources in low to middle-low resource countries. Two major reasons are the lack of recognition of critical care’s value to the overall health system and inadequate funding for critical care. It is essential to recognise that critical care reinforces the entire hospital system and the health system overall, and empowering healthcare professionals to deliver comprehensive critical care can reduce high mortality and morbidity rates. This study focuses on a low-resourced country, examining all levels of ICU and specialties.

It is essential to recognise that interprofessional care in the ICU can be physically and emotionally demanding due to the patient’s critical condition, the management of family emotions, and the treatment decisions made by the ICU team that significantly influence patient outcomes. The ICU team must consistently demonstrate excellent interprofessional communication by sharing clear information, critically evaluating their words and actions, and consistently showing strong clinical reasoning. They should utilise data from physical assessments, advanced ICU technologies, and diagnostic tests as a foundation for clinical decision-making. Additionally, ethical decisions should be carefully formulated to prevent dilemmas while fostering effective collaboration in addressing life-threatening situations and managing team dynamics that can arise during critical moments of moral judgment. Ultimately, these key factors in interprofessional care promote a safe patient environment in the ICU, creating a positive psychological atmosphere that reduces adverse events and helps lower mortality and morbidity rates. Furthermore, improving interprofessional care enhances team cooperation and cohesion, which helps decrease the occurrence of incivility among ICU team members.

In 2008, the World Health Organization recommended a triple purpose for improving the health system. The three main goals are (1) to improve population health, (2) to raise the quality of patient care, and (3) to reduce healthcare system costs. Brodenheimer and Sinky recently updated the proposal, supporting a quadruple-aim approach that highlights the importance of enhancing the well-being and satisfaction of healthcare teams, especially considering the high rates of attrition and burnout among healthcare professionals. This emphasises the need to strengthen strong, interconnected healthcare teams, especially in intensive care units, which directly affect the quality and expense of healthcare. Since strengthening interprofessional care teams is vital, and ICU teams are highly interconnected, healthcare education must incorporate components that prepare professionals to demonstrate effective interprofessional skills. Each person can gain knowledge from, with, and about others to provide safe, high-quality care for critically ill patients. Literature shows progress in integrating interprofessional education in industrialised countries. However, in developing countries, especially in Sub-Saharan Africa, the integration of interprofessional education is still in its early stages. It requires a gradual incorporation into healthcare curricula to familiarise practitioners with interprofessional skills early in their careers.^9,10^

Consequently, the absence of integrative interprofessional education in most healthcare professional curricula results in most ICU team members being predominantly taught in isolation before licensing, with no exposure to interprofessional education.^11^ The fragmented structure of health professions education results in deficiencies in the necessary interprofessional competencies for effective collaboration within ICU teams, impacting information sharing, decision-making, values and ethics, comprehension of patient safety principles, team performance, and conflict resolution. The identified gaps result in conflicting interventions that increase the likelihood of adverse outcomes in the ICU.^12,13^ Moreover, evidence indicates that ICU patients frequently succumb to the consequences of inadequate interprofessional collaboration among ICU team members.^14-16^ Consequently, initiatives to promote interprofessional collaboration are vital for establishing a safe culture and enhancing the performance of ICU teams.

**Figure 1.**
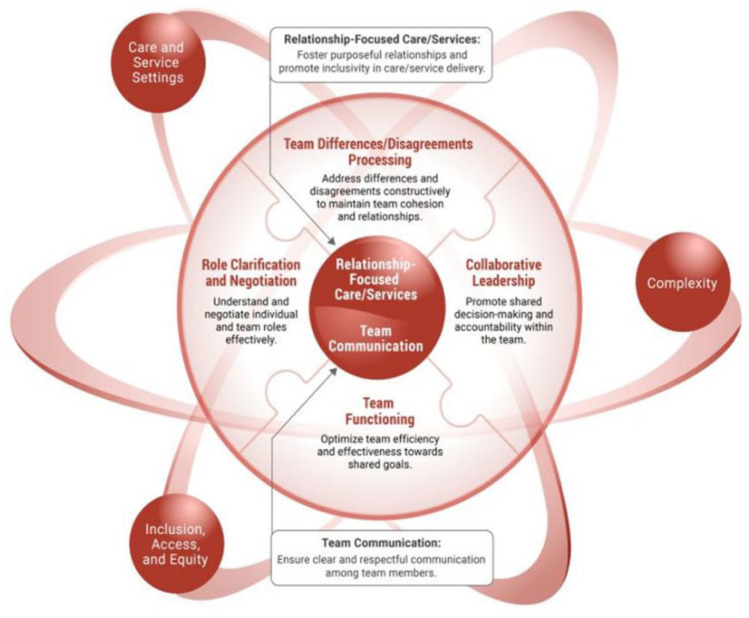
CIHC competency framework for advancing collaboration 2024. Source: Canadian Interprofessional Health Collaborative, www.cihc-cpis.com.

The National Interprofessional Competency Framework was established by the Canadian Interprofessional Health Collaborative in 2010 to strengthen interprofessional education and collaborative practices in the ICU and other healthcare fields.^17^ In 2016, the Canadian Interprofessional Health Collaborative (CIHC) board revised the competency frameworks to encompass four competency domains: the first is the Values and ethics for interprofessional work that foster an environment of mutual respect and shared principles. Secondly, the roles and duties foster an understanding of individual roles and those of other professions to effectively evaluate and handle patients’ healthcare requirements and enhance community health. Thirdly, interprofessional communication facilitates productive interactions with patients and among professionals, fostering a responsive and accountable dialogue that enhances a collaborative approach to health promotion, maintenance, and illness prevention and treatment. The fourth aspect is Teams/Teamwork, emphasising relationship-building ideals and the concepts of team dynamics necessary for optimal performance in various team positions. The objective is to strategise, implement, and assess patient- and population-centred care, along with health programmes and policies that are safe, timely, efficient, effective, and equitable. This study utilised the 2016 framework as a reference for the survey questions. After examining the survey results, the CIHC revised the interprofessional competency domain framework in 2024. The study’s results corroborated the revised competency domains and the underlying elements that facilitate the practical demonstration of interprofessional competencies by ICU teams. Refer to Figure 1 below for the new interprofessional competency framework for 2024.

The revised CIHC competency framework comprises six competency domains essential for ICU teams to facilitate practical interprofessional collaborative activities. Within the ICU team framework, the first of these six domains is relationship-focused care, which cultivates intentional relationships among the ICU team and enhances inclusivity in the provision of critical care. The second domain is team communication, which fosters straightforward and courteous interactions among ICU teams. The third competency domain is role clarification and negotiation, which enhances comprehension of responsibilities within the ICU environment and facilitates effective negotiation with various persons and teams. The fourth area pertains to processing team differences and disagreements, which fosters the capacity to manage conflicts and sustain coherence and relationships within ICU teams. The fifth competency domain is team functioning, promoting ICU teams to exhibit efficiency and effectiveness in pursuing common objectives. The sixth competency domain is collaborative leadership, necessitating ICU teams to show collaborative leadership by engaging in shared decision-making and collective accountability.^17^ The six competency domains underscore the knowledge, abilities, attitudes, and values characterising the behaviours and beliefs essential for interprofessional collaborative activities.

The revised interprofessional competency framework includes key considerations that are essential to understanding and implementing the framework. These considerations involve Access, Inclusion, and Equity, emphasizing that ICU team members should be aware of diversity and socioeconomic factors that can influence their interactions and demonstration of interprofessional competencies. It is important to address the complexities of the ICU environment, which can range from stratified to highly complex. The ICU environment is inherently intricate due to the nature of the patient population and the significant reliance on interprofessional teamwork. Additionally, the aspects of care and environment are fundamental considerations. In an ICU setting, this includes the prevalent circumstances, governing protocols and guidelines, communication models used by the team, decision-making processes, and the involvement of non-healthcare stakeholders, such as chaplains, in addressing patients’ spiritual needs. Limited research has been conducted in the Sub-Saharan region on the impact of interprofessional competencies within ICUs. In developed countries, studies show that integrating interprofessional education (IPE) into the traditional curriculum is a strategic approach to managing human resource shortages. However, fewer studies have explored how improving interprofessional competencies in ICUs can enhance practical teamwork to address challenges faced by low-resource countries. South Africa, as a Sub-Saharan nation, requires the identification of key interprofessional competencies that enable ICU teams to practice collaboratively and effectively within its specific context.

Integrating IPE in the traditional curriculum is a cornerstone for its sustainability and cost-effectiveness in low to middle-income countries [14], which can help IPE adoption even in resource-constrained countries. However, due to limited evidence from developing countries, these guidelines were based on evidence derived from developed countries. The paradigm shift in the epidemiological transition in lower and middle-income countries necessitates a few health workers from various disciplines to work together to address the pertinent global health challenges.

Furthermore, according to the African Interprofessional Education Network (AfrIPEN), there is limited evidence regarding interprofessional education and collaboration in lower-resource settings, including Sub-Saharan African countries.^10^ This is despite the greater need in the region, where there is a higher prevalence of limited resources, disease burden, poverty, and workforce shortages common challenges that lead to poor healthcare collaboration and less effective service delivery.^10^ North West Province (NWP) in South Africa has faced a crisis for many years. The Ritshidze report^18^ highlights a persistent and widespread stockout crisis, with ICUs frequently running out of essential supplies and medicines, leaving patients without access to necessary treatment or care. Additionally, there is a shortage of human resources across the province, especially in rural areas, which results in people being sent home without proper assessment and treatment. Despite the importance of interprofessional collaborative efforts to ensure access to safe, quality care in rural NWP, to the authors’ knowledge, no research has been conducted to assess the interprofessional collaboration competencies among ICU teams in the North West Province. This study aims to identify the interprofessional skills needed for ICU teams in NWP to promote effective collaboration.

## Problem Statement

The North West Province in South Africa is one of the provinces that is currently facing significant challenges in the ICU settings. The healthcare infrastructure in the NWP is strained due to limited resources, staff shortages, and underfunding, especially within the rural areas where access to quality care is more difficult.^18,19^ Many of the ICUs lack specialised medical equipment and trained interprofessional healthcare professionals who can effectively manage critical care cases. While some districts in the NWP offer ICU interprofessional care, they are frequently overburdened and unable to meet the patient care demands and patients alongside their families. In ICUs, the patients and their families complain of a lack of key population-friendly services; there is limited access to quality healthcare services, inclusivity in care planning, respect, and a sense of safety in the ICUs.^18^ Moreover, the 2021 Ritchidze report, which conducted an audit in the NWP, showed that patients wait an average of 5:13 hours in the district hospitals to be assessed for possible admission to the ICU, and there are no defined protocols for ICU admission criteria. Although efforts to improve the ICU facilities are ongoing, the NWP continues to struggle to deliver consistent and effective interprofessional care across the diverse and often underserved critical patients.^18^ The phenomenon is an excellent concern for access to quality and patient care in ICU patients, as the indicated challenges lead to ICU healthcare professionals providing task-oriented care instead of comprehensive care to patients.^15,20^

The ICU teams must function proficiently as an interprofessional team to provide comprehensive care, exhibiting sound knowledge and technical skills to work in a team, and interpersonal skills relative to the ICU to interact effectively with the ICU team.^19,20^ However, in the NWP in 2019 to 2021, the province lost R200 million in litigation cases, which were linked to poor interprofessional collaboration, interprofessional communication, inadequate skills in managing team dynamics, long waiting times to be assessed and cared for, and a lack of reporting practices among the ICU teams.^21^ That phenomenon led to misdiagnosis and late patient care intervention.^21^ Moreover, the regulatory bodies, such as the South African Nursing Council (SANC), South African Pharmacy Council (SAPC), and Health Professional Council of South Africa (HPCSA), have limited, clearly defined expectations for the development and sustainability of sound interprofessional competencies among the ICU healthcare professionals.^9,20,22^ That is reflected in the formal undergraduate training curricula in the three statutory bodies.^15,22^ The inclusion of interprofessional competencies training is still at its infancy stage in South Africa, NWP, with a significant number of registered ICU healthcare professional who has no pre- and post-licensure training in interprofessional competencies and their importance for safe and quality patient care.^1,9,20,22^ Moreover, the accredited continuous professional development programmes (CPDs) activities across the indicated three statutory bodies vary from hospital to hospital, or service providers, and do not necessarily include interprofessional collaborative care practices (IPCP) knowledge, skills, and attitude required for healthcare professionals within the ICU teams.^23^ Nor are the CPD activities that promote ICU teams to learn from, with, and about each other in healthcare settings such as NWP, where there is a gross lack of resources.^20^ This phenomenon exposes the ICU teams without IPCP competencies to error, creates professional tension between ICU team members increases conflicts and opportunities for compromised patient safety.^23^ Considering the above discussion, it will be of value to determine the interprofessional competencies that the ICU teams in the NWP regard as critical for effective interprofessional collaborative care to enhance awareness of interprofessional competencies of ICU teams to mitigate the heightened litigation cases.

## Methods

The study employed a cross-sectional survey methodology to examine interprofessional abilities among ICU specialists in the North West region of South Africa to enhance interprofessional collaboration practices.^24^ The cross-sectional survey approach was employed to collect data from a substantial sample size within a defined timeframe concerning the interprofessional abilities necessary for ICU teams. The research population comprises ICU professionals in the North-West Province of South Africa. Furthermore, stratified random sampling was employed to guarantee that each unit in the population had an equal probability of selection, hence ensuring representativeness and precision of estimations.^25^

### Ethical Considerations

The Health Research Ethical Committee at North-West University accepted the study and assigned it a unique ethical number (NWU-0051-23-A1). Subsequently, approval was granted for the goodwill permission requested by the four Research Gatekeeper Committees representing two public and two private hospitals. The independent research assistants who were the clinical facilitators in the four selected ICUs distributed informed consent paperwork for participants to complete, confirming their voluntary participation in the study without compulsion. Written informed consent was obtained from all the participants by the independent research assistants and saved in an encrypted file according to the higher learning institution’s data management policy.^24^ For transparency of the study, the researcher compiled a further information video clip. The information video was sent to prospective respondents and included an overview of the study, the significance of the study in the NWP, its aims and goals, and the contact details of the lead researcher if they had any questions to ensure transparency.

### Population

The target population required for the study to answer the research question was the nurses, doctors, clinical facilitators, and pharmacists who were working in intensive care units in four hospitals across the North West Province, South Africa.^25^ The indicated population was from the private and public sectors (Table 1). The total population for each of the selected professionals was the following: nurses (n = 101), doctors (n = 59), clinical facilitators (n = 4), and pharmacists (n = 4). The study featured a unique ethnic mixture which included blacks, coloureds, whites, and Indian participants for an inclusive perception on the topic. The respondents were not selected based on their ethnicity, but they were willing to participate in the study.

**Table 1.** Number of Items Under Each Competency Domain.

Scale	Total items
Values/ethics for interprofessional practice	V10
Roles/responsibilities	RR10
Interprofessional communication	CC8
Teams and teamwork	TT10

Source: Core Interprofessional competencies: Core competencies for interprofessional collaborative practice, 2016. Press. https://ipec.memberclicks.net/assets/2016-Update.pdf.

### Sampling and Sample Size

A stratified sampling method was employed to categorise the population into distinct groups, ensuring each individual belonged to a particular stratum. The diverse professional jobs among the targeted population necessitated the implementation of stratified sampling.^25^ Furthermore, there existed four hospitals: two private and two public. Each hospital was considered a stratum to ensure participation from all facilities. Moreover, the researchers selected the participants based on their relevant experience, knowledge, and qualities in an interprofessional collaborative environment within the ICU to address the research question: “What interprofessional competencies are necessary in the ICU for effective interprofessional collaboration?” The sample size was 88

#### Inclusion Criteria

- ICU nurses have over 2 years of experience in an ICU setting, whether employed on a permanent, contractual, or flexible basis. Additionally, retired nurses had over 2 years of expertise in the ICU. The nurses were selected due to their involvement in the ICU team, their execution of care prescribed by the interprofessional team, and their understanding of interprofessional teamwork within the ICU. Physicians with over 2 years of experience in the ICU or retired physicians possessing expertise in ICU team dynamics are anticipated to collaborate with the ICU team to provide high-quality patient care.
- Pharmacists with a minimum of 2 years of experience in the ICU. The pharmacists collaborate with doctors and nurses to ensure appropriate care for critically ill patients. They guarantee medication availability in the ICU and assess the proper administration of medications, among other responsibilities.
- ICU clinical facilitators with permanent or contract positions and over 2 years of experience in the ICU, providing training for the ICU team and comprehending the dynamics of ICU teams and the nature of interprofessional collaboration.
- The ICU healthcare professionals willingly engage in the study and provide informed consent.
- The retired nurses and doctors were selected because, even though they are not actively practicing, they have experiential wisdom from years of working in the ICU. They understand the ICU team dynamics and the team expectations for quality critical care.
- The overall sample size of the population was 88.

### Exclusion Criteria

Nurses, doctors, pharmacists, and clinical facilitators without experience working in the intensive care units, as they will not be able to answer the research question.

### Study Setting

The research was carried out in the public and private healthcare sectors of the North West Province, South Africa. A considerable proportion of ICU nurses and physicians lack postgraduate training in critical care, which qualifies them as specialists in the ICU. The absence of postgraduate training in ICU care further constrains the delivery of high-quality, crucial care in the ICU.^26^ Consequently, the researcher aimed to enhance awareness of interprofessional capabilities within the ICU teams to address the issues of restricted staffing resources.

### Research Instrument

The data was gathered with a 5-point Likert scale instrument, with responses ranging from 1 (strongly disagree) to 5 (strongly agree). The study instrument with items for each component was derived from the interprofessional competency domain framework. The instrument has been employed in multiple studies about intensive care.^15,27,28^ The research instrument had 43 items measured at nominal or ordinal levels. The questionnaire comprised five sections that assessed different themes. The questionnaire is organised around four fundamental competencies established by the Canadian Interprofessional Health Collaborative: values and ethics for interprofessional practice, roles and responsibilities, interprofessional communication, and cooperation. Each questionnaire segment addresses particular facets of these competencies, yielding significant insights into the ethical standards, role clarity, communication efficacy, and collaborative dynamics among ICU personnel.

The initial segment of the questionnaire comprised informed consent, allowing respondents to indicate their agreement or disagreement to participate in the study, followed by inquiries aimed at collecting demographic data, encompassing variables such as gender, age, experience, and role in the ICU. Section 2 analysed values and ethics pertinent to interprofessional practice. Section 3 assessed the roles or duties of the participants. Section 4 analysed issues of interprofessional communication, whereas Section 5 scrutinised teams and teamwork. See Table 1 with the items on each competency domain.

### Pilot Study

A pilot study was performed with 30 enumerators selected from the study area. The pilot study aimed to evaluate the questionnaire’s effectiveness and identify any obstacles respondents may encounter while completing it. The questionnaire was piloted to assess the face validity and clarity of the items. The piloting was conducted using a limited convenience sample of participants. The data was analysed by a statistician and incorporated into the study’s overall findings. The respondents who participated in the pilot study were also included in the final data.

### Data Collection

An official letter granting ethical approval was disseminated to the participants to enable data collection from the selected responders. Additionally, we incorporated an opening video that clarified the study’s aims. The video was used as a form of marketing and recruitment to the ICU professionals with assistance from the clinical facilitators and the doctor’s relations officer. The principal instrument for data collection was a self-administered questionnaire disseminated to ICU personnel in the North-West Province of South Africa. The self-administered questionnaire was employed to gather data from ICU professionals in the North-West Province between December 1, 2023, and June 29, 2024. Participation was optional. The participants were ensured secrecy and anonymity. One hundred sixty-eight structured questionnaires were randomly disseminated to ICU professional nurses, medical doctors, pharmacists, and clinical training facilitators. Eighty-eight questionnaires were accurately completed and collected, yielding a 52% response rate.

### Reliability of the Data Collection Tool

Maree and Pietersen^29^ Define dependability as the degree to which a tool yields consistent results across multiple occasions or varied samples from the same population. The Canadian Interprofessional Health Collaborative established and verified the Interprofessional Collaborative Framework for Advancing Collaboration.^16,30^ Researchers have employed questionnaires in ICU studies, yielding reliable results that validate the essential competencies for interprofessional collaborative practices in ICUs across Canada, Europe, the United States, and South Africa.^16,31^ Subsequently, following the survey’s data collection, the statistician employed Cronbach’s Alpha to validate the construct of the questions, ensuring their consistency and the likelihood of obtaining similar responses from participants.

### Data Management

The data was exported to Statistical Package for the Social Sciences software (SPSS)for statistical analysis. The researcher, with the statistician, checked the quality of the obtained data, and cleaning was done, where the names of the respondents were removed. The missing values were deleted as per the missing data thumb rule.

### Data Analysis

The data collected from the responses were analysed using SPSS version 29.0. The results include descriptive statistics in the form of graphs, cross-tabulations, and other figures for the quantitative data collected. Inferential techniques employed in this analysis include correlations, chi-square test, Kruskal-Wallis test, and exploratory factor analysis (EFA)

## Results

### Section A: Demographic Data

Table 2 presents the demographic characteristics of the participants included in the study.

**Table 2.** The Table Below Describes the Data.

Role in ICU	Frequency/numbers	Percentage
ICU professional nurse	68	77.3
ICU medical doctor	11	12.5
ICU pharmacist	4	4.5
ICU clinical training facilitator	5	5.7
*Age (category) (years)*		
20-29	12	13.6
30-39	31	35.2
40-49	25	28.4
50-59	17	19.3
60-69	3	3.4
*Gender*		
Female	83	94.3
Male	5	5.7
*Years of experience (category)*		
<10	54	61.4
10-20	23	26.1
>20	11	12.5

Source: Authors own work.

### Competency Domain 1: Values/Ethics for Interprofessional Practice

Examining the data within this skill category demonstrates uniformly high ratings across all categories, signifying a robust commitment to values and ethics among ICU teams (Table 3). The average scores for all items exceed 4.65, with the highest average score of 4.89 recorded for “Respect the dignity and privacy of patients while maintaining confidentiality in the delivery of team-based care” (VE2). This indicates that ICU staff rank the preservation of patient confidentiality and privacy, which is a core ethical component in healthcare. The minimal standard deviations, between 0.32 and 0.54, signify a limited degree of diversity in the responses, implying that the respondents predominantly concur on the significance of these ethical standards. The median score for all items is 5.00, reaffirming the strong consensus among respondents. The binomial test *P*-values are all below .001, signifying that the observed frequencies deviate from the predicted frequencies under the assumption of random guessing, with a threshold score of 3.0. This robust consensus underscores the dedication of ICU teams to maintaining elevated ethical standards in their practice. The ramifications of these findings for the development and execution of interprofessional education and training programmes are significant. The robust commitment to ethical standards within ICU teams establishes a firm basis for fostering interprofessional collaboration and enhancing patient care results. It also indicates that future training initiatives may strengthen these principles while tackling new ethical dilemmas in interprofessional practice.

**Table 3.** Values/Ethics for Interprofessional Practice Results.

Competency Domain 1: values/ethics for interprofessional practice		Strongly agree (5)/%	Agree (4) /%	Neural (3) /%	Disagree (2) /%	Strongly disagree (1) /%	Mean	Standard deviation	Relative importance index
VE2	Respect the dignity and privacy of patients while maintaining confidentiality in the delivery of team-based care	88.5	11.5	0.0	0.0	0.0	4.9	0.32	97.7
VE10	Maintain competence in one’s profession appropriate to the scope of practice	81.4	17.4	0.0	1.2	0.0	4.8	0.49	95.81
VE9	Act with honesty and integrity in relationships with patients, families, communities, and other team members	79.3	18.4	2.3	0.0	0.0	4.8	0.47	95.4
VE3	Embrace the cultural diversity and individual differences that characterise patients, populations, and the health team	76.1	23.9	0.0	0.0	0.0	4.8	0.43	95.23
VE4	Respect the unique cultures, values, roles/responsibilities, and expertise of other health professions and the impact these factors can have on health outcomes	78.4	19.3	2.3	0.0	0.0	4.8	0.48	95.23
VE6	Develop a trusting relationship with patients, families, and other team members (CIHC, 2010)	78.4	19.3	2.3	0.0	0.0	4.8	0.48	95.23
VE5	Work in cooperation with those who receive care, those who provide care, and others who contribute to or support the delivery of prevention and health services and programmes	75.0	25.0	0.0	0.0	0.0	4.8	0.44	95
VE7	Demonstrate high standards of ethical conduct and quality of care in contributions to team-based care	71.6	27.3	1.1	0.0	0.0	4.7	0.48	94.09
VE1	Place the interests of patients and populations at the centre of inter-professional health care delivery and population health programmes and policies, to promote health and health equity across the lifespan	71.6	25.0	3.4	0.0	0.0	4.7	0.54	93.64
VE8	Manage ethical dilemmas specific to inter-professional patient/population-centred care situations	65.9	33.0	1.1	0.0	0.0	4.7	0.5	92.95

Source: Interprofessional competency domain 1: Core competencies for interprofessional collaborative practice, 2016. Press. https://ipec.memberclicks.net/assets/2016-Update.pdf.

### Competency Domain 2: Roles/Responsibilities

The research revealed that ICU teams typically assess their comprehension and execution of tasks and responsibilities favourably. The item “Communicate with team members to clarify each member’s responsibility in executing components of a treatment plan or public health intervention” (RR6) received the highest mean score of 4.71 and a low standard deviation of 0.55, indicating robust consensus and uniformity among respondents, as seen in Table 4. Each item’s elevated median scores (all 5.00) reinforce this agreement. The statement “Forge interdependent relationships with other professions within and outside of the health system to improve care and advance learning” (RR7) received the lowest mean score of 4.17 and the highest standard deviation of 1.03, signifying increased response variability. This indicates that although most respondents acknowledge the significance of interprofessional interactions, problems or variances in experiences may influence their perspectives. The persistently low *P*-values (<.001) from the binomial test validate that most respondents assessed these competencies markedly above the neutral threshold of 3.0. This robust consensus underscores the importance of open communication, acknowledging limitations, involving different specialists, and fostering continual professional development within ICU teams. The findings indicate that ICU teams are adequately equipped for effective collaboration. However, there exists potential to improve interprofessional interactions and learning further. These results can guide focused initiatives and training programmes to enhance interprofessional collaboration and improve patient care outcomes.

**Table 4.** Competency Domain 2: Roles/Responsibilities Results.

Competency Domain 2: roles/responsibilities		Strongly agree (5)/%	Agree (4) /%	Neural (3) /%	Disagree (2) /%	Strongly disagree (1) /%	Mean	Standard deviation	Relative importance index (%)
RR6	Communicate with team members to clarify each member’s responsibility in executing components of a treatment plan or public health intervention	74.7	23.0	1.1	1.1	0.0	4.7	0.6	94.3
RR8	Engage in continuous professional and inter-professional development to enhance team performance and collaboration	72.4	23.0	4.6	0.0	0.0	4.7	0.6	93.6
RR5	Use the full scope of knowledge, skills, and abilities of professionals from health and other fields to provide care that is safe, timely, efficient, effective, and equitable	67.8	29.9	1.1	1.1	0.0	4.6	0.6	92.9
RR2	Recognise one’s limitations in skills, knowledge, and abilities	66.7	29.9	2.3	1.1	0.0	4.6	0.6	92.4
RR1	Communicate one’s roles and responsibilities clearly to patients, families, community members, and other professionals	68.2	26.1	3.4	1.1	1.1	4.6	0.7	91.8
RR3	Engage diverse professionals who complement one’s professional expertise, as well as associated resources, to develop strategies to meet specific health and healthcare needs of patients and populations	60.9	36.8	2.3	0.0	0.0	4.6	0.5	91.7
RR9	Use the unique and complementary abilities of all members of the team to optimise health and patient care	64.4	31.0	2.3	2.3	0.0	4.6	0.7	91.5
RR4	Explain the roles and responsibilities of other providers and how the team works together to provide care, promote health, and prevent disease	60.9	35.6	2.3	1.1	0.0	4.6	0.6	91.3
RR10	Describe how professionals in health and other fields can collaborate and integrate clinical care and public health interventions to optimise population health	57.0	34.9	7.0	1.2	0.0	4.5	0.7	89.5
RR7	Forge interdependent relationships with other professions within and outside of the health system to improve care and advance learning	48.3	32.2	10.3	6.9	2.3	4.2	1.0	83.5

Source: Interprofessional competency domain 2: Core competencies for interprofessional collaborative practice 2016. Press. https://ipec.memberclicks.net/assets/2016-Update.pdf.

### Competency Domain 3: Interprofessional Communication

The research demonstrated uniformly elevated scores across all categories, signifying robust consensus among respondents regarding the significance of successful interprofessional communication. Table 6 shows the average scores for all items to surpass 4.45, with the highest average of 4.77 for “Listen actively and encourage ideas and opinions of other team members” (CC4). This indicates that ICU teams place a high significance on active listening and recognising the contributions of team members. The item “Communicate the importance of teamwork in patient-centred care and population health programmes and policies” (CC8) has a mean score of 4.72, highlighting the significance of teamwork in communication techniques. The standard deviations vary from 0.52 to 1.04, with the item “Express one’s knowledge and opinions to team members involved in patient care and population health improvement with confidence, clarity, and respect” (CC3) demonstrating the most incredible diversity. This signifies variations in the confidence and clarity with which team members articulate their expertise and viewpoints, indicating a potential area for enhancement. The binomial test *P*-values for all items are below .001, indicating that the respondents’ evaluations are significantly above the neutral threshold of 3.0 (Tables 5 and 6).

**Table 5.** Competency Domain 3: Interprofessional Communication Results.

Competency Domain 3: interprofessional communication		Strongly agree (5)/%	Agree (4) /%	Neural (3) /%	Disagree (2) /%	Strongly disagree (1) /%	Mean	Standard deviation	Relative importance index
CC4	Listen actively and encourage the ideas and opinions of other team members	80.3	18.2	0.0	1.5	0.0	4.77	0.5	95.5
CC8	Communicate the importance of teamwork in patient-centred care and population health programmes and policies	77.4	19.4	1.6	0.0	1.6	4.72	0.7	94.2
CC6	Use respectful language appropriate for a given difficult situation, crucial conversation, or conflict	73.5	22.1	4.4	0.0	0.0	4.70	0.6	93.8
CC2	Communicate information with patients, families, community members, and health team members in an understandable form, avoiding discipline-specific terminology when possible	73.9	23.2	1.4	0.0	1.4	4.68	0.7	93.6
CC1	Choose effective communication tools and techniques, including information systems and communication technologies, to facilitate discussions and interactions that enhance team function	75.6	18.6	2.3	0.0	3.5	4.63	0.8	92.6
CC7	Recognise how one’s uniqueness (experience level, expertise, culture, power, and hierarchy within the health team) contributes to effective communication, conflict resolution, and positive inter-professional working relationships (University of Toronto, 2)	63.2	29.4	4.4	1.5	1.5	4.51	0.8	90.3
CC5	Give timely, sensitive, instructive feedback to others about their performance on the team, responding respectfully as a team member to feedback from others	62.1	28.8	3.0	4.5	1.5	4.45	0.9	89.1
CC3	Express one’s knowledge and opinions to team members involved in patient care and population health improvement with confidence, clarity, and respect, working to ensure a common understanding of information, treatment, care decisions, and population health	66.7	24.6	1.4	1.4	5.8	4.45	1.0	89.0

Source: Interprofessional competency domain 3: Core competencies for interprofessional collaborative practice 2016. Press. https://ipec.memberclicks.net/assets/2016-Update.pdf.

**Table 6.** Competency Domain 4: Teams and Teamwork Results.

Competency Domain 4: teams and teamwork		Strongly agree (5)/%	Agree (4) /%	Neural (3) /%	Disagree (2) /%	Strongly disagree (1) /%	Mean	Standard deviation	Relative importance index
TT10	Use available evidence to inform effective teamwork and team-based practices	94.2	0.0	4.3	1.4	0.0	4.9	0.5	97.4
TT4	Integrate the knowledge and experience of health and other professions to inform health and care decisions while respecting patient and community values and priorities/preferences for care	91.4	0.0	8.6	0.0	0.0	4.8	0.6	96.6
TT5	Apply leadership practices that support collaborative practice and team effectiveness	91.4	0.0	7.1	0.0	1.4	4.8	0.7	96.0
TT3	Engage health and other professionals in shared patient-centred and population-focused problem-solving	89.9	0.0	8.7	1.4	0.0	4.8	0.7	95.7
TT2	Develop consensus on the ethical principles to guide all aspects of teamwork	88.2	0.0	11.8	0.0	0.0	4.8	0.7	95.3
TT11	Perform effectively on teams and in different team roles in a variety of settings	87.5	0.0	12.5	0.0	0.0	4.8	0.7	95.0
TT8	Reflect on individual and team performance for individual as well as team performance improvement	88.6	0.0	8.6	2.9	0.0	4.7	0.7	94.9
TT9	Use process improvement to increase the effectiveness of inter-professional teamwork and team-based services, programmes, and policies	87.1	0.0	11.4	1.4	0.0	4.7	0.7	94.6
TT1	Describe the process of team development and the roles and practices of effective teams	86.0	0.0	11.6	1.2	1.2	4.7	0.8	93.7
TT6	Engage self and others to constructively manage disagreements about values, roles, goals, and actions that arise among health and other professionals and with patients, families, and community members	84.3	0.0	15.7	0.0	0.0	4.7	0.7	93.7
TT7	Share accountability with other professions, patients, and communities for outcomes relevant to prevention and healthcare	82.9	0.0	17.1	0.0	0.0	4.7	0.8	93.1

Source: Interprofessional competency 4: Core competencies for interprofessional collaborative practice 2016. Press. https://ipec.memberclicks.net/assets/2016-Update.pdf.

The statistical significance demonstrates a robust agreement among ICU teams on the essential function of excellent communication in their practice. Several trends become apparent after analysing these results about the three components found in factor analysis: Active Listening and Feedback, Knowledge and Opinion Sharing, and Effective Communication Tools and Techniques. Items about Active Listening and Feedback (CC4, CC5, CC6) exhibit elevated mean scores and minimal standard deviations, indicating a uniform consensus on the significance of these competencies. This shows that ICU teams excel in active listening and delivering polite feedback, essential for fostering a collaborative team atmosphere. The elements of Knowledge and Opinion Sharing (CC3, CC7, CC8) indicate a significant agreement on the necessity for straightforward and courteous communication of knowledge and perspectives. Nonetheless, the increased variability in item CC3 suggests that although most professionals acknowledge the significance of confidently sharing knowledge, there may be discrepancies in implementing this practice. Ultimately, the components of Effective Communication Tools and Techniques (CC1, CC2) underscore the need to employ suitable communication methods and technology. The elevated mean ratings for these categories indicate that ICU teams excel in selecting efficient communication technologies to enhance team interactions, hence providing transparent and effective information dissemination. In summary, the consensus on the significance of interprofessional communication competencies among ICU teams highlights the essential role of good communication in improving team performance and patient care. The findings from this analysis can guide focused interventions to enhance communication skills, especially in regions with greater variability, thus fostering more cohesive and successful interprofessional collaboration in ICU environments.

### Competency Domain 4: Teams and Teamwork

The data demonstrated uniformly elevated scores across all questions, signifying robust consensus among respondents regarding the significance of diverse facets of teamwork (Table 7). The average scores for all items surpass 4.66, with the highest average of 4.87 for “Utilise available evidence to inform effective teamwork and team-based practices” (TT10). This indicates that ICU teams emphasise evidence-based methods within collaborative environments. The median ratings for all items are 5.00, indicating a unified agreement on the importance of these competencies. The standard deviations vary from 0.54 to 0.82, with the item “Describe the process of team development and the roles and practices of effective teams” (TT1) exhibiting the most diversity. This suggests discrepancies in respondents’ perceptions and descriptions of team development processes, indicating a potential need for further training or clarification. The binomial test *P*-values for all items are below .001, suggesting that most respondents evaluated these competencies significantly above the neutral threshold of 3.0. This robust consensus emphasises the essential function of efficient cooperation in ICU environments and shows the collective dedication to elevated standards of team collaboration.

**Table 7.** The Median of All Groups Is Equal; At Least One Median Group Is Different from the Others.

	Null hypothesis	Test	Sig.^a,b^	Decision
1	Competency Domain 1: values/ethics for interprofessional practice are the same across categories of age (years)	Independent-samples Kruskal-Wallis Test	0.141	Retain the null hypothesis
2	Competency Domain 2: roles/responsibilities are the same across categories of age (years)	Independent-samples Kruskal-Wallis Test	0.663	Retain the null hypothesis
3	Competency Domain 3: interprofessional communication is the same across categories of age (years)	Independent-samples Kruskal-Wallis Test	0.300	Retain the null hypothesis
4	Competency Domain 4: teams and teamwork are the same across categories of age (years)	Independent-samples Kruskal-Wallis Test	0.010	Reject the null hypothesis

Source: Interprofessional Competencies: Core competencies for interprofessional collaborative practice, 2016. Press. https://ipec.memberclicks.net/assets/2016-Update.pdf.

a The significance level is 0.050.

b Asymptotic significance is displayed.

Upon investigation of the two components revealed in factor analysis—Team Dynamics and Accountability, and Leadership and Problem-Solving—various tendencies become apparent. Items about Team Dynamics and Accountability (TT1, TT2, TT4, TT6, TT7, TT9, TT10) exhibit elevated mean scores and minimal standard deviations, indicating uniform consensus on the significance of team responsibilities, ethical standards, and collective accountability. This demonstrates that ICU teams are adept at comprehending and controlling team dynamics, which are crucial for efficient collaboration and patient care. The components related to Leadership and Problem-Solving (TT3, TT5, TT8, TT11) demonstrate significant consensus, with average scores consistently exceeding 4.66. This signifies that ICU teams acknowledge the significance of leadership approaches, ongoing enhancement, and efficient problem-solving within team environments. The elevated scores for items like “Apply leadership practices that support collaborative practice and team effectiveness” (TT5) and “Engage health and other professionals in shared patient-centred and population-focused problem-solving” (TT3) underscore the significance attributed to leadership and collaborative problem-solving within ICU teams. The consensus on the importance of collaboration competencies within ICU teams highlights the essential function of excellent team dynamics and leadership in improving patient care.

### Interprofessional Collaborative Practice and Age

The trend shows that older respondents, especially those over 30, rate their competency domain in teams and teamwork significantly higher than younger respondents (20-29 years). The results indicate that individuals’ perceived ability to work within teams increases as they age, with those aged 50 to 59 showing the highest scores. See Table 7, outlining the median of all ages.

### Interprofessional Collaborative Practice and Years of Experience

Respondents with more than 20 years of experience have a significantly higher mean score for Competency Domain 1 (4.93) than those with 10 to 20 years of experience (4.68). This suggests that individuals with over 20 years of experience perceive higher values/ethics for interprofessional practice than those with 10 to 20 years of experience. Similarly, respondents with over 20 years of experience have a significantly higher mean score (4.93) than those with less than 10 years of experience (4.75). This suggests that more experienced individuals rate Competency Domain 1 higher, indicating stronger perceptions of values/ethics for interprofessional practice. Below is Table 8 with the Kruskal-Wallis test for interprofessional collaborative practice and years of experience in NWP, South Africa. Table 9 represents the Pairwise comparison of years of experience.

**Table 8.** Kruskal-Wallis Test for Interprofessional Collaborative Practice and Years of Experience.

	Null hypothesis	Test	Sig.^a,b^	Decision
1	Competency Domain 1: values/ethics for interprofessional practice are the same across categories of years of experience	Independent-samples Kruskal-Wallis Test	0.023	Reject the null hypothesis
2	Competency Domain 2: roles/responsibilities are the same across categories of years of experience	Independent-samples Kruskal-Wallis Test	0.201	Retain the null hypothesis
3	Competency Domain 3: interprofessional Communication is the same across categories of years of experience	Independent-samples Kruskal-Wallis Test	0.236	Retain the null hypothesis
4	Competency Domain 4: teams and teamwork are the same across categories of years of experience	Independent-samples Kruskal-Wallis Test	0.495	Retain the null hypothesis

Source: Interprofessional Competencies: Core competencies for interprofessional collaborative practice, 2016. Press. https://ipec.memberclicks.net/assets/2016-Update.pdf.

a The significance level is 0.050.

b Asymptotic significance is displayed.

**Table 9.** Pairwise Comparisons of Years of Experience.

Sample 1 to Sample 2	Test statistic	Std. error	Std. test statistic	Sig.	Adj. sig.^a^
10-20 to <10	5.750	6.083	0.945	0.345	1.000
10-20 to >20	−24.292	8.957	−2.712	0.007	0.020
<10 to >20	−18.542	8.082	−2.294	0.022	0.065

Source: Interprofessional Competencies: Core competencies for interprofessional collaborative practice, 2016. Press. https://ipec.memberclicks.net/assets/2016-Update.pdf.

Each row tests the null hypothesis that the Sample 1 and 2 distributions are identical. Asymptotic significances (2-sided tests) are displayed. The significance level is 0.050.

a Significance values have been adjusted by the Bonferroni correction for multiple tests.

### Interprofessional Collaborative Practice and Gender

Gender differences in the perception of Competency Domain 1 indicate that female respondents are more likely to rate their values and ethics in interprofessional practice higher than male respondents. This significant difference points to a gender-based variation in the understanding or application of values and ethics within the interprofessional context. See Table 10 for the Kruskal-Wallis Test for interprofessional practice and Gender.

**Table 10.** Kruskal-Wallis Test for Interprofessional Practice and Gender.

Hypothesis test summary				
	Null hypothesis	Test	Sig.^a,b^	Decision
1	Competency Domain 1: values/ethics for interprofessional practice are the same across categories of gender	Independent-samples Kruskal-Wallis Test	0.011	Reject the null hypothesis
2	Competency Domain 2: roles/responsibilities are the same across categories of gender	Independent-samples Kruskal-Wallis Test	0.211	Retain the null hypothesis
3	Competency Domain 3: interprofessional communication is the same across categories of gender	Independent-samples Kruskal-Wallis Test	0.445	Retain the null hypothesis
4	Competency Domain 4: teams and teamwork are the same across categories of gender	Independent-samples Kruskal-Wallis Test	0.669	Retain the null hypothesis

Source: Interprofessional Competencies: Core competencies for interprofessional collaborative practice, 2016. Press. https://ipec.memberclicks.net/assets/2016-Update.pdf.

a The significance level is 0.050.

b Asymptotic significance is displayed.

### Interprofessional Collaborative Practice and Roles in ICU

The results indicate that none of the tests yielded significant *P*-values (all are greater than .05), meaning the null hypothesis is retained for each competency domain. This implies that the distribution of responses for each competency domain (Values/Ethics, Roles/Responsibilities, Interprofessional Communication, and Teams/Teamwork) does not differ significantly across different roles in the ICU. See Table 11 below for the Kruskal-Wallis Test for interprofessional practice and roles in the ICU.

**Table 11.** Kruskal-Wallis Test for Interprofessional Practice and Roles in ICU.

	Null hypothesis	Test	Sig.^a,b^	Decision
1	Competency Domain 1: values/ethics for interprofessional practice are the same across categories of roles in the ICU	Independent-samples Kruskal-Wallis Test	0.900	Retain the null hypothesis
2	Competency Domain 2: roles/responsibilities are the same across categories of roles in the ICU	Independent-samples Kruskal-Wallis Test	0.527	Retain the null hypothesis
3	Competency Domain 3: interprofessional communication is the same across categories of roles in the ICU	Independent-samples Kruskal-Wallis Test	0.487	Retain the null hypothesis
4	Competency Domain 4: teams and teamwork are the same across categories of roles in the ICU.	Independent-samples Kruskal-Wallis Test	0.765	Retain the null hypothesis

Source: Interprofessional Competencies: Core competencies for interprofessional collaborative practice, 2016. Press. https://ipec.memberclicks.net/assets/2016-Update.pdf.

a The significance level is 0.050.

b Asymptotic significance is displayed.

## Discussion

The study provides a unique insight into the interprofessional competencies that ensure effective interprofessional collaborative practices amongst the intensive care professionals in the North West Province of South Africa. The ICU professionals who completed the online self-administered survey in this study agree to place value on all four interprofessional competencies and their importance in providing quality and safe critical care in intensive care units. These findings are significant as they reflect that the ICU professionals in NWP concur with the literature, which values IPC as a strategy of promoting patient safety by reducing adverse events that can occur due to miscommunication and a lack of shared decision-making.^10,13^ Moreover, the findings are significant as they align with the universal health coverage sustainable goal^3^ and good health and well-being.^13^ Below, the authors discuss the findings from the study.

### ICU Nurses as Key Members in the Interprofessional Team in the ICU

The biographical data from the survey indicate clear patterns for the roles, experience, age, and gender of the ICU respondents. Most responders were ICU professional nurses, comprising 77.3% of the sample, whilst ICU medical doctors, pharmacists, and clinical training facilitators accounted for 12.5%, 4.5%, and 5.7%, respectively. This distribution highlights the essential function of nurses in the ICU environment. Matlakala^32^ asserts that nurses are the predominant segment of the interprofessional team in South Africa, playing crucial roles in acute care. Kendall-Gallagher et al^33^ argue that ICU nurses occupy a pivotal role within the interprofessional team, serving as the central point of patient care by collaborating closely with the ICU team to deliver continuous surveillance, problem-solving, and decision-making for patients in the intensive care unit. Nonetheless, the South African Nursing Council (SANC) has indicated a 70% reduction of critical care nurses in South Africa from 2018 to 2021. Consequently, the insufficient number of nurses poses a risk of a disjointed interprofessional team in the intensive care unit if not appropriately addressed. Cucolo et al^34^ assert that nurses possess the requisite abilities for effective coordination and optimal functioning in interprofessional practice. Effective interprofessional collaborative practice necessitates a substantial investment of time in their work, a factor that warrants clarification. Moreover, insufficient time, resources, and staff may subject ICU nurses to excessive workloads and compromise safety and quality of care. Cucolo et al^34^ propose that fostering interprofessional competencies within the ICU aids nurses in managing emotional burdens, enhances professional communication among ICU team members, and cultivates interprofessional trust, thereby promoting a favourable perception of the complexities of nursing care in the ICU.

Impact of years of experience in demonstrating interprofessional competencies for effective interprofessional collaborative practices in the ICU.

The study reveals a notable variation in years of experience, with a median of 6 years and an interquartile range (IQR) of 3 to 16 years. Significantly, 61.4% of the participants possess less than 10 years of expertise, 26.1% have between 10 and 20 years, and 12.5% more than 20 years of experience. This indicates a comparatively inexperienced workforce, potentially affecting training and professional development requirements. These findings align with those of Goldman et al,^35^ who asserted that heightened interprofessional collaboration, facilitated by robust interprofessional competencies, is influenced by various factors, including years of practice in an ICU environment. Given their few years of experience, ICU personnel are likely to encounter challenges in managing conflict, navigating hierarchical relationships, facilitating interprofessional communication, addressing seniority issues, clarifying professional roles, responding to urgency, and resolving arguments regarding end-of-life decisions. These variables are supported by a lack of understanding regarding the information, skills, beliefs, and behaviours necessary for effective collaboration within an ICU team, which is only developed via years of experience in that environment. Rotz and Duenas^36^ Performed a qualitative study examining the collaborative readiness of medical and pharmacy students aged 22 to 26 who were new to the ICU environment. The study indicated that their age impeded collaborative activities, and the findings subsequently guided the revision of their curricula to emphasise interprofessional competencies. Sulistyowati and Walker^37^ assert that the brevity of experience in the ICU affects the effective exhibition of interprofessional connections. This arises from the varying perceptions and comprehension of individuals with limited experience concerning interprofessional collaborative practice, which fosters professional dominance, divergent thought processes regarding the team-based care approach in the ICU, and dedication to patient care.

The ICU team must recognise, develop, and implement practical interprofessional skills to bridge the gap in collaborative practices caused by limited experience.^35^ The Benner Novice to Expert theory highlights that progressing through developmental levels is essential for gaining expertise, which requires a deeper understanding to identify patterns, understand expectations, engage with the culture through experience, and pursue ongoing professional growth.^38^ It is important to emphasise that the study shows highly experienced or expert individuals are leaving the young ICU workforce. These seasoned ICU specialists have built strong relationships within the ICU and offer valuable guidance and practices based on years of experience, which reduces the number of experienced ICU professionals available to mentor newly licensed ICU practitioners. This trend increases the risk of adverse events, as Novice and emerging ICU staff are still learning to manage high-acuity cases and complex situations, interventions.^37^ Additionally, novice and emerging ICU professionals may experience psychological and professional strain from a lack of mentorship. They might feel overwhelmed by the workload, fear being inadequate, and studies have shown that high turnover among ICU professionals is linked to a lack of mentorship, onboarding, and unrealistic expectations.^35,37,38^ The study indicated that 61% of ICU team members had less than 10 years of experience, while only 12.5% had more than 20 years of expertise. The limited experience hampers awareness and effectiveness in demonstrating interprofessional skills, as individuals are still developing their professional identity and understanding how to foster an interprofessional culture. It is important to recognise that professional development involves activities across interconnected areas, including professionalism, identity formation, and growth. Personal factors such as values, morals, beliefs, self-efficacy, life experiences, language, and relationships influence this process, which requires patience and guidance. Therefore, to build an interprofessional identity, the ICU team needs support through comprehensive, ongoing professional development programmes that nurture both professional and interprofessional identities in practice. Interprofessional identity involves establishing a strong cognitive, psychological, and emotional connection with an interprofessional community essential for achieving shared, context-specific goals.^39^

Influence of gender imbalance in the demonstration of interprofessional competencies in the ICU for effective interprofessional collaborative practice.

The survey showed a significantly higher number of females than males in the ICU, with both genders perceiving ethical standards differently. For males, the mean = 4.39, 95% CI: 3.82-4.96, and for females, the mean = 4.77, 95% CI: 4.70-4.85. This notable gender disparity highlights the prominence of women in ICU roles within this sample, reflecting broader trends in the nursing field. This aligns with the dominance of females in the nursing profession. A quantitative descriptive study conducted in the United States indicated that male healthcare workers, as a minority in the ICU, experience role tension in that environment. The Sherrod Role Strain Scale assessed role strain related to role conflict, role overload, role incongruity, and role ambiguity.^40^ However,^41^ also revealed that power dynamics across genders are common in ICUs. Further, it was noted that although females are the majority in the ICU, they are more likely to face bullying because they are perceived to have less power due to social patriarchal beliefs. Therefore,^42^ emphasises that if role strain and gender biases in the ICU go unrecognised and unaddressed, it can lead to poor interprofessional collaboration and incivility. The ICU team must be trained on gender equality and bias. Moreover, the team should be encouraged to identify and eliminate implicit and subconscious gender biases to foster a safe environment for effective collaboration. Additionally, diversifying the code of conduct policies can help eliminate inequality and improve inclusion in the ICU.

### Influence of Age in Demonstrating Interprofessional Competencies and Measures to Close the Gap

The study indicates that older ICU practitioners with more experience rank patient safety and value professional development in the ICU more than their younger counterparts. This enhances the fortification of team dynamics and understanding of roles, patterns, and systems within team-based care in the ICU. This strength can be improved by ongoing professional development programmes tailored for ICU teams, wherein they tackle ethical dilemmas or practice modifications via interprofessional communication and collaboration. The results are similar to those of A pre- and post-intervention study performed in six ICUs, which advocated for the ICU team’s establishment of proactive, team-oriented ethics protocols. The study promoted regular implementation of protocols to address ethical concerns, the support needed by patients and families, and the care objectives. This discourse may occur in a simulated environment to protect patients.^43^ ICU teams are presented with case scenarios to resolve collaboratively or during interprofessional rounds that incorporate external professionals, such as chaplains, particularly in palliative or end-of-life care situations.^43^ In the context of ICU teams, daily interprofessional rounds integrate the internationally recognised ICU Liberation framework, which encompasses Assessment, Prevention, and Management. Pain management for spontaneous awakening and breathing trials regarding analgesia and sedation; D for delirium assessment, prevention, and management; E for early mobility and exercise; and F for family participation and empowerment bundle. The (ABCDEF) bundle facilitates and enhances the coordination of interprofessional rounds.^44,45^ The interprofessional team rounds in the ICU promote inclusive, ethical, and supportive conduct aligned with interprofessional collaboration in the ICU. The discourse on patient care among healthcare professionals enhances ethical, patient-centred, and goal-oriented practices, aligning with the interprofessional competency domain of values and ethics for interprofessional practice.^44,45^

Furthermore, multidisciplinary rounds involve structured team-based conversations that incorporate several professional specialities collaborating to examine and manage critically ill patients. The multidisciplinary rounds enhance holistic patient care, enabling coordinated decision-making that produces favourable patient outcomes.^46^ The ICU team’s debriefing sessions with patients incorporate ethics consultations as a strategy to be addressed alongside interprofessional rounds. The specified discussion strategies enhance the interprofessional competency domain of ethics and values within ICU team consultations, mitigating patient and family distress and dissatisfaction with care while fostering trust between patients and the ICU team, ultimately promoting high-quality, patient-centred care.^46^ Furthermore, training in relational skills among ICU teams has been recognised as enhancing awareness of the interdependence of roles within the ICU, aimed at reducing power disparities and inequality while fostering social exchange and reciprocity among healthcare professionals in the ICU environment. During these relational skills training sessions, the team is urged to be authentic, transparent, attuned to emotions, and to eschew compulsion.^47^ Furthermore, employing team-based instruction on trauma-informed care encompasses safety, trustworthiness, transparency, peer support, collaboration, mutuality, empowerment, self-advocacy within a team, recognition, and considerations of cultural and gender issues.^47^ Training in trauma-informed care enhances ethical behaviours in the ICU, fosters interdisciplinary collaboration, and develops the competencies necessary for patient-family-centred care in this environment.

### Competency Domain 1: Values and Ethics for Interprofessional Collaborative Practices and Factors That Influence the Competency Domain Demonstration by the ICU Team

The study indicated that within the initial interprofessional competency domain, specifically “Values and Ethics for Interprofessional Practice,” the ICU team ranks patient safety and confidentiality, fostering optimism for safe and quality care in the intensive care unit. Intensive care units are prone to errors that might lead to negative results due to multitasking, information overload, and frequently disruptive workflows.^48^ Thus, patient safety remains a massive issue in intensive care units with global challenges, including poor reporting of adverse events, the limited number of qualified healthcare professionals in practice, and the emergence of complex diseases.^49^ ICU experts have indicated that maintaining strong ethical standards fosters a common mental framework in clinical practice, enhancing communication and heightening awareness of potential safety hazards. Ellee Edgar et al^50^ assert that for ICU teams to improve decision-making consistency and quality, they must possess shared mental models. Consequently, for effective mental models, the team must satisfy the following criteria: the ICU team must have an accurate representation of the current reality within the ICU, their decisions must be grounded in valid evidence, the ICU team must reach a consensus regarding the team objective in this case, the patient treatment plan and delineate how they will collaborate to accomplish this objective. If the specified requirements are not met, the ICU team must address and resolve any differing viewpoints that hinder the establishment of a shared mental model. Ellee Edgar et al^50^ further elucidate that the team’s comprehension and frequent communication on their work can enhance the criteria through team training and collective awareness.

### Competency Domain 2: Roles and Responsibilities and the Gaps Identified in Demonstrating the Competency Domain Amongst the ICU Teams

The study indicated that ICU teams hold in regard the second interprofessional competency domain, which examines distinct roles and responsibilities. Nevertheless, a deficiency exists in guaranteeing that all ICU teams acknowledge and uphold the various tasks, attributable to insufficient advancement in interdependent connections within the ICU team. Noce et al^51^ asserted that interdependent relationships in ICU environments rely on communication, cooperation, unity, respect, collaboration, and mutual assistance, enhancing ICU teamwork. Noce et al^51^ assert that when ICU teams integrate the relevant features with their operational methods and, crucially, their objectives, it enhances the quality of care. These traits are predicated on functionally interdependent relationships that acknowledge each other’s roles, strengths, and limitations. Mahvar et al^52^ assert that interdependent connections are essential in the ICU since they influence therapeutic efficacy. Treatment efficacy is affected by the dynamics of clinical interactions within ICU teams.

Additionally, the work environment in ICUs is characterised by critical situations and emergencies that necessitate rapid decision-making and implementation, which demands robust interdependent relationships wherein team members recognise and appreciate each other’s expertise. Consequently, interactive educational interventions may be employed to enhance the interdependent relationships within ICU teams. Comparative cohort research conducted by George et al^53^ demonstrated that interactive interprofessional simulation training offers ICU teams practical experience and facilitates cooperation and integration with an understanding of team members’ roles and duties. The interactive interprofessional simulation interventions enhance collaboration and foster cohesive behaviours among ICU teams. An interactive interprofessional simulation training effort was implemented in a surgical trauma burns unit. A notable enhancement was observed in team skills metrics, understanding of team roles and duties, retention of acquired information, and learnt teamwork abilities. Moreover, the study advocated for regular interactive team training to enhance the interdependent connections inside the ICU.^53^

### Competency Domain 3: Interprofessional Communication and How the ICU Team Perceives and Demonstrates the Competency Domain

The study has indicated that ICU teams acknowledge the third interprofessional skill domain focused on communication. The ICU team considers active listening, ongoing feedback, knowledge, and opinion exchange essential competencies in the ICU environment. Effective interprofessional communication in the ICU is essential, as precise and efficient communication is a prerequisite for high-quality care.^54^ The study indicates that the ICU team regards communication regarding teamwork in patient care, health programmes, policies, and guidelines as crucial for maintaining interprofessional communication skills. The results are corroborated by Wang et al,^54^ who assert that effective interprofessional communication in the ICU positively influences the quality of patient care and should be implemented throughout patient care and the care procedures. An integrative literature review by Wang et al^54^ examined strategies to enhance interprofessional communication in the ICU, recommending the implementation of team training for ICU personnel to facilitate mutual learning regarding the care provided in that environment. These encompass training programmes such as TeamsSTEPPS. TeamSTEPPS is an evidence-based training programme that enhances quality and safety by promoting good teamwork and communication skills in ICUs and other healthcare fields.^55^ Wang et al^54^ recommended the implementation of communication checklists, tools, and electronic SBAR templates. SBAR stands for Situation, Background, Assessment, and Recommendation. The ICU scenario pertains to the patient’s present status and the provisional diagnosis. The background in ICU pertains to the history of the presenting illness, the patient’s medical history, and their pharmaceutical regimen. Assessment in the ICU pertains to the evaluator’s appraisal of the illness or condition. The recommendation for the ICU relates to the care plan and expected alterations in condition or treatment.^54^ The proposed solutions would enhance the interprofessional competency that examines communication among the ICU team.

The ICU team indicated that expertise in managing team dynamics, leadership, and problem-solving within the ICU relies on effective interprofessional communication. Consequently, it is essential to promote leadership techniques that foster collaboration and enhance team effectiveness, enabling ICU teams to exchange strategies for delivering patient-centred or problem-based care. Yamamoto^56^ corroborates the study’s findings in a cross-sectional analysis conducted in Japan. Yamamoto^56^ asserts that leadership methods in the ICU are essential. The leadership techniques encourage the ICU team to collaborate, effectively coordinate their responsibilities, and respond swiftly to deliver critical care that addresses the requirements of patients and their families. Consequently, leadership training initiatives yield advantageous outcomes for healthcare personnel in the ICU and foster open communication and teamwork. The author contends that possessing good leadership abilities is essential for establishing relationships vital to fostering successful interprofessional collaboration practices in the ICU.^56^

### Competency Domain 4: Teams and Teamwork and How the ICU Team Demonstrates Competency in the ICU

The survey indicated that ICU practitioners unanimously recognise the significance of all facets of cooperation within the ICU, particularly team-based procedures. Salas et al^57^ characterise team-based approaches as a collaborative methodology wherein intensive care teams with complementary knowledge, abilities, and values collaborate to attain common objectives, resolve intricate issues, and make collective decisions. Salas et al^57^ assert that team-based methods are founded on essential characteristics including communication, cooperation, accountability, teamwork, adaptability, problem-solving, and interdependence. Dietz et al^47^ corroborate the study’s findings, affirming that team-based methods are essential for delivering comprehensive care in the ICU. Furthermore, the intricate nature of patient care in critical care necessitates team-based procedures to guarantee optimal treatment through coordinated efforts, varied skills, and collaborative decision-making. Jepkosgei et al^58^ confirm that team-based practices in the ICU enhance well-being and job satisfaction among the ICU staff. In contrast, inadequate team-based practices correlate with increased missed treatment, medical errors, and adverse events. The study also demonstrated that the ICU teams utilise existing evidence to enhance collaboration. This encompasses comprehending the responsibilities of each team member, implementing optimal communication methods among the many professions engaged in patient care, and utilising established collaboration strategies. Ervin et al^59^ agree with the findings and assert that the basis of effective teams and quality healthcare in intensive care relies on well-defined roles and responsibilities, efficient interprofessional communication, and team communication. Ervin et al^59^ assert that rounds constitute the formal daily face-to-face sessions attended by physicians and nurses directly engaged in patient care in ICU settings. During rounds, the ICU team deliberates at the bedside regarding patient care, progress, and potential modifications to enhance outcomes. The rounds facilitate the team process, offering the ICU team context for information sharing and collaborative decision-making. They also serve as a forum for discussing sensitive matters related to patient and family communication and strategies for managing challenging situations in the ICU. Another exemplary communication strategy employed in the ICU is the utilisation of checklists and guidelines, which mitigate obstacles to informal and formal collaboration. The checklists facilitate effective communication among ICU teams, utilising them and protocols as a systematic approach to discuss essential treatment plans, optimise care for timely reporting, and offer a framework for interactions that establish common objectives.^59^

Moreover, the study has demonstrated that the ICU staff value shared accountability in their practice within the ICU. Nevertheless, how they share culpability was not expressly examined by the questionnaire completed by the ICU staff in the study. Pellikka et al^60^ conducted a scoping review that examines shared accountability in the intensive care unit, emphasising the inclusion of patients and their families in the decision-making process. This approach fosters an environment of mutual trust and respect between ICU teams and patients or their families. Recognition of various skills, consideration of the patient’s values and beliefs in treatment planning, and open dialogue regarding the therapy process.

### Limitations

The study focused solely on nurses, doctors, and pharmacists working in the ICU, excluding those with less than 2 years of experience and other allied health professionals such as dietitians and physiotherapists. This limitation reduced the diversity of viewpoints among the interprofessional team members in the ICU. Therefore, the findings may not provide a comprehensive report of the entire ICU team. Another point to consider is that the study was conducted within a single province and did not include other provinces in South Africa. As a result, the perspectives cannot be generalised to other care areas, nor do they clarify how South African ICU teams develop interprofessional competencies for effective teamwork within the ICU. Additionally, the questionnaire was self-administered, which introduces the possibility of response bias from the ICU professionals who completed it. Respondents might have given answers that reflect what they think is socially acceptable in the ICU, rather than truly expressing their views. This could lead to an overestimation of their perception and expression of each skill domain covered in the survey. Furthermore, the survey lacked a qualitative component for open-ended responses to gather alternative viewpoints. It used a Likert scale, and without face-to-face interviews, responses were likely superficial, with participants choosing options like Agree, Strongly Agree, Disagree, or Strongly Disagree. Moreover, only 88 participants completed the survey, representing a 52% response rate, which limits the voices of the rest of the ICU team. The psychometric analysis shows strong internal reliability and structural validity, especially for VE, RR, and TT domains. The multidimensionality found in CC is understandable given the complexity of communication in interprofessional settings. However, while the tool is statistically reliable, its use and results should be interpreted with caution, considering the limited participant diversity, possible response biases, and the specific context of the study.

## Recommendations

Based on these study findings, a set of targeted recommendations is proposed to address the promotion of interprofessional competencies within the ICU teams in the NWP, South Africa.

### Health Science Education

Integrating interprofessional education and collaborative practices can facilitate transformative learning and strengthen health systems within NWP ICU teams to enhance positive health outcomes and promote cost-effective healthcare.^9^ Consequently, the study advocates for incorporating interprofessional team-based education among healthcare professionals in South Africa, enabling them to learn collaboratively about diverse subjects. The interprofessional team-based learning will demonstrate interprofessional teamwork within the ICU team, aligning with one of the competency domains outlined in the interprofessional competency framework.^61^ The fundamental concepts of interprofessional team-based learning encompass mandated out-of-class preparation by health professional educators. This subsequently motivates health science faculties to deliver training on interprofessional team-based learning for optimal implementation. Furthermore, interprofessional team-based learning incorporates in-class team assessments, fostering critical thinking and collaborative problem-solving, skills that healthcare professionals are expected to exhibit in practice. Consideration may be given to topics such as the ethics of care, clinical care in the ICU, and overall emergency management situations through interprofessional simulation-based training. Interprofessional simulation-based learning allows healthcare professional students to use theoretical knowledge in practice and reflect on their own and their colleagues’ performance through debriefing. Furthermore, interprofessional team-based learning is crucial for enhancing knowledge, confidence, and collaboration.^62^

### Clinical Practice

NWP ICU teams can be motivated to collaboratively evaluate ICU clinical guidelines and protocols collaboratively, enabling them to assess the efficiency and effectiveness of these guidelines for better patient outcomes. Additionally, NWP ICU teams can systematically incorporate interprofessional rounds into patient care to improve shared decision-making by utilizing expertise and collaborative problem-solving. This approach will promote a comprehensive, patient-centred methodology in the ICU. Furthermore, improving interprofessional collaborative practices also addresses challenges healthcare systems face, including the shortage of healthcare personnel and the rising costs from litigation due to adverse events and negligence.^63-72^

### Collaboration Between Clinical Settings and Higher Learning Institutions for Capacity Building

Consequently, clinical educators might collaborate with faculty of affiliated higher education institutions within the NWP to create interprofessional ongoing professional development programmes that reinforce interdependent relationships among ICU teams. Nevertheless, previous situational analyses conducted in NWP ICU environments regarding the issues faced or through surveys will ascertain the substance of the CPD programmes to address the needs of the ICU workers. Consequently, the CPD programmes can be utilised to tackle patient safety concerns within NWP ICU environments, employing ethical situations that arise in the NWP ICU for realistic team-based problem-solving.

### Embracing Interprofessional Continuous Professional Development Programmes

The CPD programmes can use an interactive facilitative method in which the facilitators possess expertise, represent all participants, and foster collaborative learning among the participants. Employ critical thinking, problem-solving, and collaborative leadership; they are anticipated to engage in dialogue and provide mutual feedback throughout the process. Courses like Team Strategies and Tools to Enhance Performance and Patient Safety (Team STEPPS), which mitigate ICU medical errors, augment team awareness, delineate team roles, resolve conflicts, improve information sharing, and elevate the quality of care and patient safety, can be regarded as instrumental in enhancing the interprofessional competencies of ICU teams.

### Recommended Studies to Explore

Additional study may be undertaken employing diverse research approaches to investigate further and elucidate the subsequent questions that the survey could not address, as the is limited literature conducted in the North West Province (NWP). The following research can be conducted:

- What is the influence of role strain in the interprofessional collaborative practices in the NWP ICU and its contribution to safe and quality care?
- What contributes to poor interdependence relationships in the NWP ICU?
- What are the implications of delayed development of interprofessional identity in patient care within the NWP ICU?
- How do NWP ICU teams measure interprofessional team performance, and how is the feedback used as evidence-based practice for safe and quality care in the ICU?
- How do interprofessional NWP ICU teams share accountability in the ICU?
- What is the impact of effective role clarification in ensuring quality and safe practice in the NWP ICU?
- How can a shared mental model be developed in NWP ICU teams for effective interprofessional collaborative practices in the ICU?
- What is the effective leadership style that can promote effective interprofessional collaborative practices in the NWP ICU?

## Conclusion

All-inclusive interprofessional competencies within the ICU are very important for collaborative practices, according to the ICU teams in the North West Province, South Africa. However, values and ethics for interprofessional care, along with teamwork in the ICU, are considered more important by the ICU teams in North West Province, South Africa. Interprofessional competencies connect and promote synergy and positive patient outcomes when demonstrated competently by the ICU teams.
